# Cannabidiol has a unique effect on global brain activity: a pharmacological, functional MRI study in awake mice

**DOI:** 10.1186/s12967-021-02891-6

**Published:** 2021-05-24

**Authors:** Aymen H. Sadaka, Ana G. Ozuna, Richard J. Ortiz, Praveen Kulkarni, Clare T. Johnson, Heather B. Bradshaw, Bruce S. Cushing, Ai-Ling Li, Andrea G. Hohmann, Craig F. Ferris

**Affiliations:** 1 Center for Translational NeuroImaging, Northeastern University, Boston, MA USA; 2 Department of Biological Sciences, University of Texas At El Paso, El Paso, TX 79968 USA; 3 Psychological and Brain Sciences, Program in Neuroscience, Indiana University, Bloomington, IN USA; 4 Gill Center for Biomolecular Science, Indiana University, Bloomington, IN USA; 5 Psychology and Pharmaceutical Sciences, Northeastern University, Boston, MA USA; 6 Department of Psychology, Northeastern University, 125 NI Hall, 360 Huntington Ave, Boston, MA 02115-5000 USA

**Keywords:** Tonic immobility, Behavioral arrest, Reticular activating system, Olfaction, *N*-acyl-phosphatidylethanolamines-specific phospholipase D, PTSD, Negative BOLD

## Abstract

**Background:**

The phytocannabinoid cannabidiol (CBD) exhibits anxiolytic activity and has been promoted as a potential treatment for post-traumatic stress disorders. How does CBD interact with the brain to alter behavior? We hypothesized that CBD would produce a dose-dependent reduction in brain activity and functional coupling in neural circuitry associated with fear and defense.

**Methods:**

During the scanning session awake mice were given vehicle or CBD (3, 10, or 30 mg/kg I.P.) and imaged for 10 min post treatment. Mice were also treated with the 10 mg/kg dose of CBD and imaged 1 h later for resting state BOLD functional connectivity (rsFC). Imaging data were registered to a 3D MRI mouse atlas providing site-specific information on 138 different brain areas. Blood samples were collected for CBD measurements.

**Results:**

CBD produced a dose-dependent polarization of activation along the rostral-caudal axis of the brain. The olfactory bulb and prefrontal cortex showed an increase in positive BOLD whereas the brainstem and cerebellum showed a decrease in BOLD signal. This negative BOLD affected many areas connected to the ascending reticular activating system (ARAS). The ARAS was decoupled to much of the brain but was hyperconnected to the olfactory system and prefrontal cortex.

**Conclusion:**

The CBD-induced decrease in ARAS activity is consistent with an emerging literature suggesting that CBD reduces autonomic arousal under conditions of emotional and physical stress.

**Supplementary Information:**

The online version contains supplementary material available at 10.1186/s12967-021-02891-6.

## Introduction

CBD has anxiolytic properties, reducing the autonomic and emotional responses to stress and interfering with the consolidation and extinction of fearful memories [1], which has been associated with anxiety disorders [2], autism spectrum disorder [3], psychosis [4] and post-traumatic stress disorder [5]. It’s potential as a therapeutic compound is emphasized by the fact that CBD is the primary active compound in the anti-epileptic drug, Epidiolex [6]. CBD has a complex pharmacology within the brain impacting multiple receptors by altering the lipidome, increasing and/or decreasing lipid mediators in specific brain areas [7], associated with dose, neurological condition and the environment. The primary targets for CBD given systemically are unknown. Non-invasive magnetic resonance imaging (MRI) using changes in BOLD (blood oxygen level dependent) signal has been used to detect the immediate increases and decreases in site-specific brain activity in response to various drugs [8–11]. The changes in BOLD signal are basically a proxy for increases and decreases in cerebral blood flow to areas of increased and decreased metabolic activity, respectively. Several studies in humans have used functional BOLD imaging to look at the neuroanatomy affected by treatment with CBD [12–19]. These studies looking at the effects of CBD have all evaluated a single oral dose given prior to scanning. While this approach establishes a baseline of resting state blood flow that changes with different task-related paradigms or differs from placebo or healthy controls in response to a preexisting condition, they do not address the effects of repeated exposure or the potential for dose-dependent changes in activity, consistent with drug target specificity.

Pharmacological MRI (phMRI) is a non-invasive method to evaluate neural circuitry involved in the behavioral effects of drugs independent of their specific biochemical mechanism [20]. To our knowledge, no published reports, in either animals or humans, have used phMRI to assess the immediate dose-dependent effects of CBD on global brain activity. Therefore, the goal of the present study was to characterize the dose-dependent changes in brain activity induced by CBD. Given that CBD may have a narrow dose range, impact multiple targets, and show context-dependent efficacy, phMRI is an ideal method to globally assess the integrated effects of CBD across multiple neural circuits to understand how CDB may impact anxiety and fear. We predict that at a certain dose there would be a decrease in relative activity, assessed using BOLD, in neural circuitry controlling stress-related behaviors. To test our prediction, we imaged awake mice using three different doses of CBD.

## Methods

### Animal usage

Male C57BL/J6 mice (n = 60), ages 100–120 days, weighing between 28–30 g, were obtained from Charles River Laboratories (Wilmington, Massachusetts, USA). While a majority of phMRI studies have been conducted in rats [21], we chose to study mice based on previous work from our group on CBD induced changes in *N*-acyl-phosphatidylethanolamines-specific phospholipase D (NAPE-PLD) activity [22]. Mice were maintained on a 12:12 h light–dark cycle with lights on at 07:00 h and allowed access to food and water ad libitum. All mice were acquired and cared for in accordance with the guidelines published in the Guide for the Care and Use of Laboratory Animals (National Institutes of Health Publications No. 85–23, Revised 1985) and adhered to the National Institutes of Health and the American Association for Laboratory Animal Science guidelines. The protocols used in this study complied with the regulations of the Institutional Animal Care and Use Committee at the Northeastern University and adhered to the ARRIVE guidelines for reporting in vivo experiments in animal research [23].

### Drug preparation and administration

CBD was a gift from the Center for Drug Discovery (Northeastern University, Boston MA) and dissolved in EtOH/cremophor/saline 1:1:18 for I.P. injections. Following acclimation, mice were randomly assigned to one of four groups corresponding to EtOH/cremophor/saline vehicle, 3, 10, or 30 mg/kg I.P. CBD. The amount of drug was adjusted to deliver vehicle and each dose in a volume of 0.2 ml. To deliver drug remotely during the imaging session, a poly-ethylene tube (PE-20), approximately 30 cm in length, was positioned in the peritoneal cavity. The range of doses of CBD evaluated were taken from the literature [24–26].

### Awake mouse imaging

#### Imaging system

We used previously described awake mouse imaging techniques [27]. Briefly, we used a quadrature transmit/receive volume coil customized for optimal space filling, anatomical resolution, and signal-to-noise. The mouse holder (Ekam Imaging; Boston, MA) fully stabilizes the head in a cushioned helmet, minimizing discomfort caused by ear bars and other restraint systems that are commonly used to immobilize the head for awake animal imaging. A movie showing the set-up of a mouse for awake imaging is available at http://www.youtube.com/watch?v=W5Jup13isqw. The effectiveness of this passive restraining system can be judged by the minimal level of motion artifact recorded during the imaging session as shown in Additional file 1: Figure S1. The average displacement in any orthogonal direction over the entire 15 min scanning session did not exceed 56 µm.

#### Acclimation

A week before imaging, mice were acclimated to the head restraint and the noise of the scanner [27]. The acclimation protocol was repeated over four consecutive days reducing autonomic nervous system-induced effects during awake animal imaging (e.g., changes in heart rate, respiration, corticosteroid levels and motor movements), to improve contrast-to-noise ratios and image quality [28]. Only mice that habituate to restraint were used in the analysis. Additionally, three mice died and five were lost to motion artifact or technical complications resulting in group sizes of EtOH/cremophor/saline vehicle (n = 8), 3 mg/kg (n = 6), 10 mg/kg (n = 5), and 30 mg/kg (n = 7).

#### BOLD phMRI and pulse sequence

Experiments were conducted using a Bruker Biospec 7.0T/20-cm USR horizontal magnet (Bruker; Billerica, MA) and a 20-G/cm magnetic field gradient insert (ID = 12 cm) capable of a 120-µsec rise time. At the beginning of each imaging session, a high-resolution anatomical data set was collected using the rapid acquisition relaxation enhanced (RARE) pulse sequence (18 slices; 0.75 mm; field of view (FOV) 1.8 cm; data matrix 128 × 128; time to repeat (TR) 2.1 s; time to echo (TE) 12.4 ms; Effective TE 48 ms; number of averages (NEX) 6; 6.5 min acquisition time). Functional images were acquired using a multi-slice Half Fourier Acquisition Single Shot Turbo Spin Echo (HASTE) pulse sequence (18 slices; 0.75 mm; FOV 1.8 cm; data matrix 96 × 96; TR 6 s; TE 4 ms; Effect ET 24 ms; 15 min acquisition time; in-plane resolution 187.5 µm^2^). Spin echo is required to achieve the high anatomical fidelity required for data registration to the mouse MRI atlas as shown in Additional file 1: Figure S2 [29]. Each functional imaging session consisted of uninterrupted data whole brain scans, 150 scan repetitions, total elapsed time 15 min. The control window included the first 50 scan repetitions, a 5 min baseline. Following the control window, an I.P. injection of drug was given followed by a 10 min stimulation window consisting of acquisitions 50–150.

The dose-dependent effect of CBD on brain activity was quantified by measuring positive and negative percent changes in BOLD signal relative to baseline as previously described [30]. A complete description of the data analysis is provided in Additional file 1: phMRI analysis.

#### Resting state functional connectivity

Sixty min prior to imaging mice were injected I.P. with EtOH/cremophor/saline vehicle (n = 10) or 10 mg/kg CBD (n = 10). The mice were then anesthetized and fitted into the coil as described above. Mice were maintained under light 1% isoflurane anesthesia (ambient air mix), adjusted to hold the respiratory rate between 50–60 breaths/min as compared to a normal rate of 85–90 breaths/min. Scans were collected using a spin-echo triple-shot EPI sequence (imaging parameters: matrix size = 96 × 96 × 20 (H × W × D), TR/TE = 1000/15 ms, voxel size = 0.312 × 0.312, slice thickness = 1.2 mm, with 200 repetitions, total time 10 min. The data processing, normalization and group level analysis is described in detail in Additional file 1**.**

### Resting state BOLD functional connectivity analysis

#### Degree centrality

All network analysis was computed with Gephi, an open-source network analysis and visualization software [31]. Absolute values of the CBD and vehicle symmetric connectivity matrices were imported, and edges were loaded as undirected networks. A complete description of the graph theory analysis is provided in Additional file 1: Graph Theory Analysis.

#### CBD analysis

To validate that there was a dose-dependent change in CBD levels, serum levels of cannabinoids were analyzed [22]. In brief, methanolic extracts of 90 µl of serum were partially purified on C18 solid phase extraction columns (Zorbax) and eluants were analyzed using HPLC/MS/MS (API 3000, Applied Biosystems). Deuterium-labeled anandamide elutes in the same fraction as CBD and was used as an internal standard to monitor recovery. Levels of CBD and THC were analyzed using standard curves with Analyst Software as previously described [22]. During analysis it was discovered that each of the samples contained a small fraction THC in addition to CBD. This can occur during synthesis and is often unknown if the levels are not analyzed. The plasma ratio in each dose was *ca* 25:1 CBD:THC (Additional file 1: Figure S3B).

## Results

**Fig. 1:**
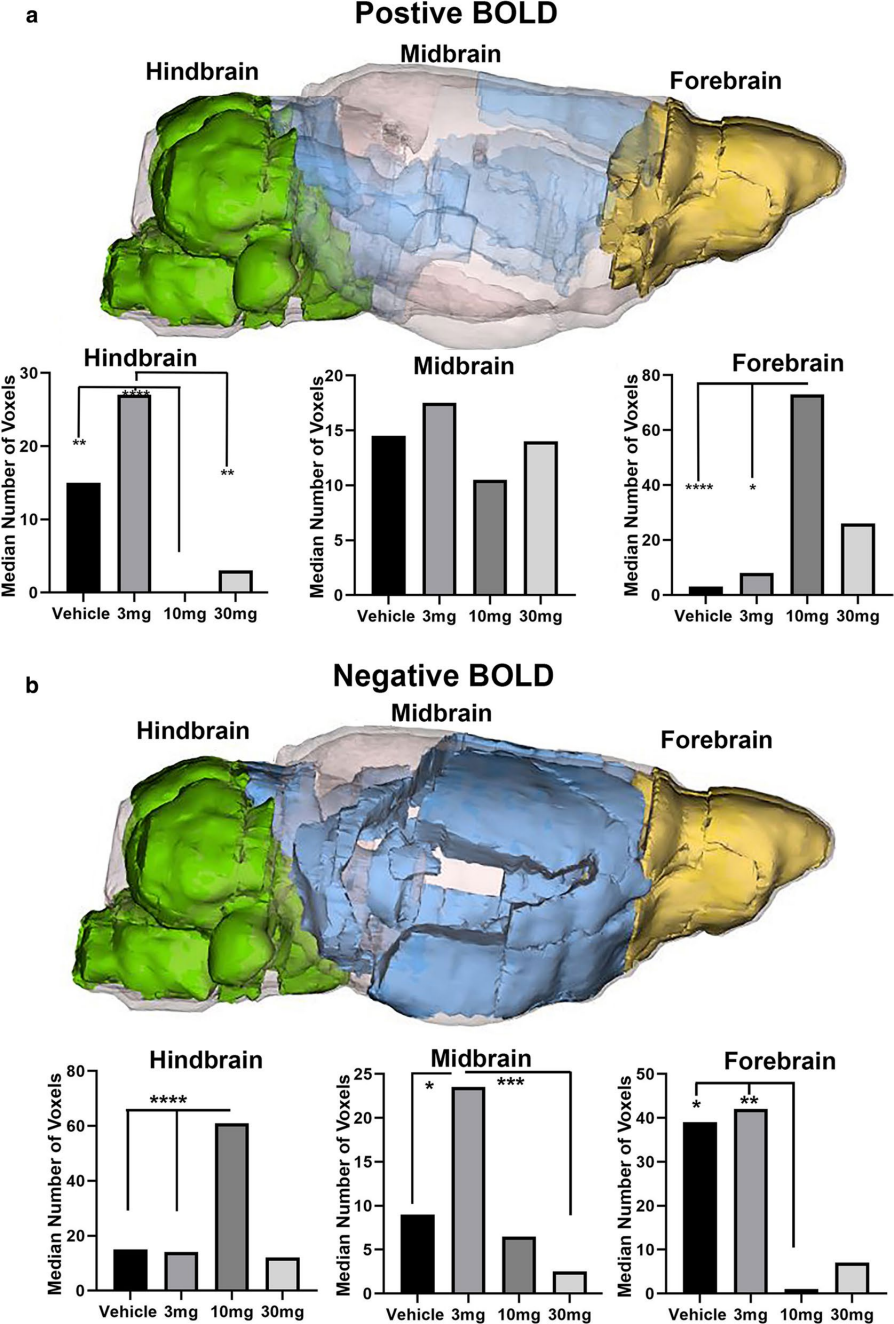
Polarized Positive and Negative BOLD. The color-coded 3D reconstructions for positive and negative BOLD denote the location of the brain areas comprising the hindbrain, midbrain, and forebrain, respectively. The bar graphs below show the average median number of voxels from each of these brain regions for vehicle, 3, 10, and 30 mg/kg I.P. doses of CBD. For forebrain positive BOLD: (****p < 0.0001, 10 mg > Veh); (*p = 0.0133, 10 mg > 3 mg). For hindbrain positive BOLD: (**p = 0.0012, 10 mg < Veh); (****p < 0.00001, 10 mg < 3 mg); (**p = 0.0019, 10 mg < 3 mg). For forebrain negative BOLD: (*p = 0.0197, 10 mg < Veh); (**p = 0.0043 10 < 3 mg). For midbrain negative BOLD: (*p = 0.0389; 3 mg > Veh); (***p = 0.0006, 3 mg > 30 mg). For hindbrain negative BOLD:D voxels (median ca 16) that is significantly increased with the 10 mg dose of CBD over vehicle and 3 mg (****p ≤ 0.0001, 10 mg > Veh, 10 mg > 3 mg)

Shown in Table 1 is a truncated list of 35 out of 138 brain areas ranked in order of their significance for change in positive BOLD volume of activation (number of voxels). Reported is the median number of voxels significantly activated 10 min post injection of vehicle (Veh), 3, 10 and 30 mg/kg of CBD with a critical value α < 0.05. Shown are p values and effect size (omega square ω^2^) calculations for each brain area. Additional file 1: Table S1 for positive BOLD volume of activation is provided for all 138 brain areas. Areas highlighted in gold show an inverted U-shape dose response as commonly reported in the literature with the 10 mg/kg dose which produced a mean plasma concentration of 115 mg/ml (Additional file 1: Figure S3) being most effective [24–26]. Note that these areas are associated with the olfactory bulb (e.g., glomerular layer, granular layer) and prefrontal cortex (e.g., frontal association, orbital, infralimbic and prelimbic cortices) as shown in Fig. 1. In contrast, many of the areas highlighted in green show a U-shaped dose response (e.g., flocculus cerebellum, crus ansiform lobule, pontine area with the 3 mg dose being most effective and the 10 mg least effective. There are no brain areas that show a stepwise dose-dependent increase in brain activity. The areas highlighted in green are all located in hindbrain, brainstem, and cerebellum (see Fig. 1). The areas highlighted in blue, all of which have some of the lowest effect sizes, show both U- and inverted U-shaped responses and are located between the forebrain and hindbrain areas.

**Table 1:**
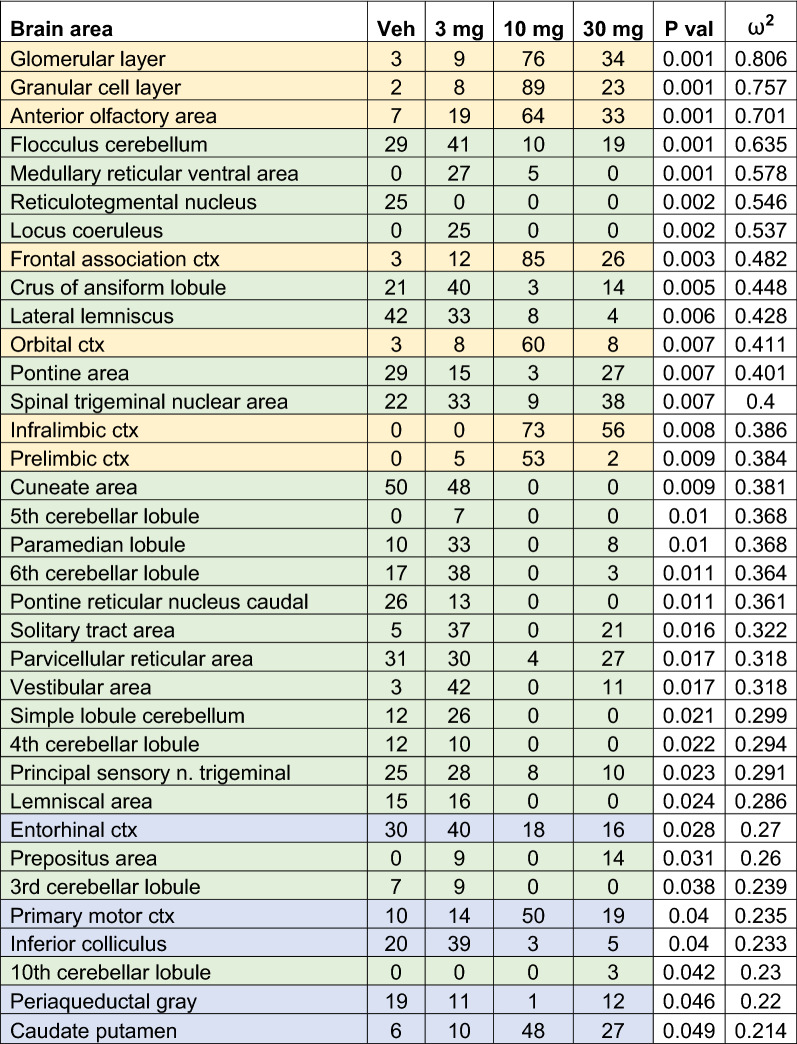
Positive bold volume of activation

This table is a truncated list of 35 out of 138 brain areas ranked in order of their significance for change in positive BOLD volume of activation (number of voxels). Reported is the median number of voxels significantly activated 10 min post injection of vehicle (0), 3, 10 and 30 mg/kg I.P. doses of CBD. Show are p values and effect size (omega square ω^2^) for each brain area. The significance levels for FDR was p ≤ 0.051. Areas highlighted in gold are associated with the olfactory system and prefrontal cortex. Areas highlighted in green are in the hindbrain brainstem and cerebellum. The areas highlighted in blue are located between the forebrain and hindbrain areas

Shown in Table 2 is a truncated list of 50 out of 138 brain areas ranked in order of their significance for change in negative BOLD volume of activation. A false discovery rate for multi-comparisons gives a significance level of p ≤ 0.073. The areas highlighted in gold are again the olfactory bulb and prefrontal cortex, as in Table 1, but the pattern is reversed, with areas like the glomerular layer and orbital cortex showing a U-shaped dose response. The 3 mg/kg dose is most effective in causing a negative change in BOLD signal while the 10 mg/kg dose is least effective. This reversed pattern between positive and negative BOLD is also true for the areas highlighted in green representing the hindbrain, brainstem, and cerebellum. The 10 mg/kg dose is most effective in causing a negative change in BOLD signal with many areas presenting with the inverted U-shaped dose response. The brain areas highlighted in blue are located along the rostral/caudal axis between the forebrain and hindbrain (Fig. 1).

**Table 2:**
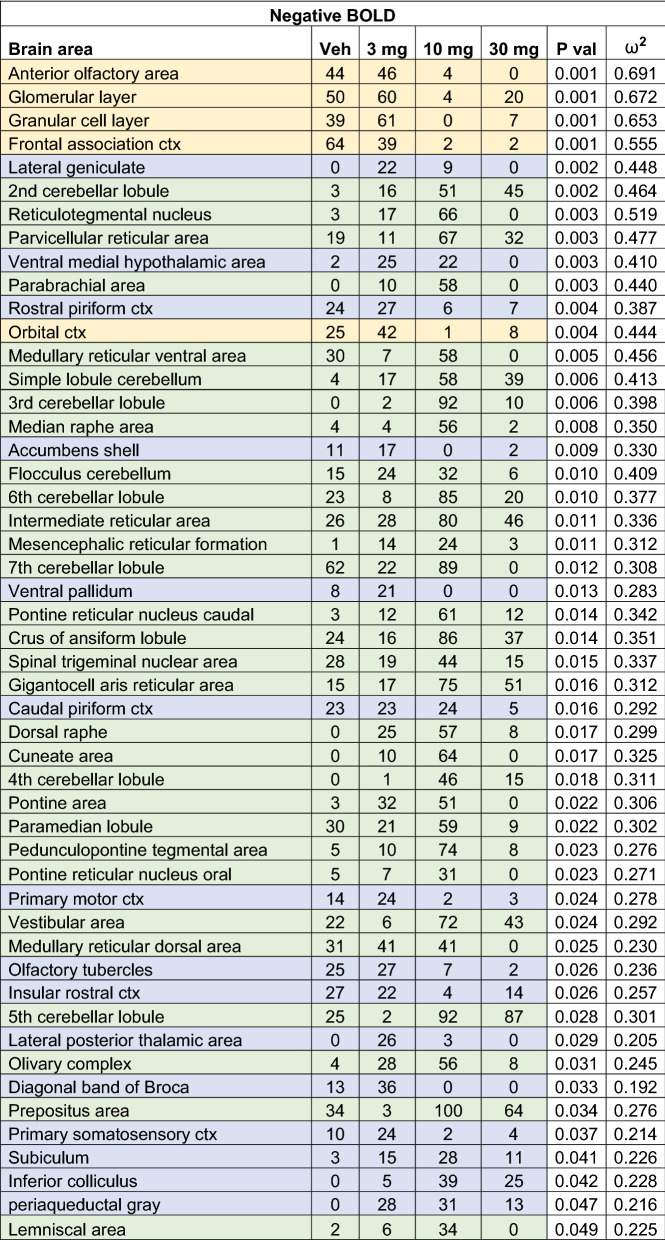
Negative BOLD volume of activation

This Table is a truncated list of 50 out of 138 brain areas ranked in order of their significance for change in negative BOLD volume of activation. Reported is the median number of voxels significantly activated 10 min post injection of vehicle (0), 3, 10 and 30 mg/kg I.P. doses of CBD. Show are p values and effect size (omega square ω^2^) for each brain area. The significance level for FDR was p ≤ 0.073. The areas highlighted in gold are the olfactory bulb and prefrontal cortex. Areas highlighted in green are localized to the hindbrain brainstem, and cerebellum. The brain areas highlighted in blue are located along the rostral/caudal axis between the forebrain and hindbrain

The data from Tables 1 & 2 are shown in the respective bar graphs in Fig. 1. In forebrain, the average of all medians scores from areas in gold for positive BOLD volume of activation, was higher in the 10 mg/kg group compared to either vehicle (p < 0.0001) or the 3 mg/kg (p = 0.0133) (Fig. 1a). There were no significant differences between doses for the midbrain areas. In the hindbrain, the average of all median scores from areas in blue for positive BOLD, volume of activation for CBD (10 mg/kg) was lower than that observed following vehicle (p = 0.0012). The median number of voxels for 3 mg/kg was greater than both 10 mg/kg (p < 0.00001) and 30 mg/kg (p = 0.0019). There was no difference in voxel activation in the hindbrain between vehicle and 3 mg/kg. There is a baseline of voxel activation in the brainstem/cerebellar areas in awake mouse imaging that is reduced by 10 and 30 mg/kg doses.

This relationship between CBD doses and positive BOLD was reversed for negative BOLD (Fig. 1b). Unlike positive BOLD areas highlighted in gold, olfactory bulb, and prefrontal cortex, show a U-shaped dose response. The 3 mg/kg the most effective in causing a negative change in BOLD signal while 10 mg/kg was least effective. This reversed pattern between positive and negative BOLD is also true with the hindbrain brainstem, and cerebellum showing an inverted U-shape, with 10 mg/kg dose stimulating the strongest negative BOLD signal (Fig. 1b). In the forebrain, there is a baseline of negative BOLD voxels (median ca 40) that is significantly reduced with either 10 mg/kg (p = 0.0197) or 3 mg/kg CBD (p = 0.0043) versus vehicle. In the hindbrain, the baseline of negative BOLD voxels (median ca 16) was increased with both 10 mg/kg and 3 mg/kg CBD (p ≤ 0.0001 for each comparison) over vehicle. In the midbrain brain areas, 3 mg/kg increased the median number of negative voxels over vehicle (p = 0.0389), whereas 30 mg/kg was associated with a lower number of negative voxels than the 3 mg/kg (p = 0.0006).

**Fig. 2:**
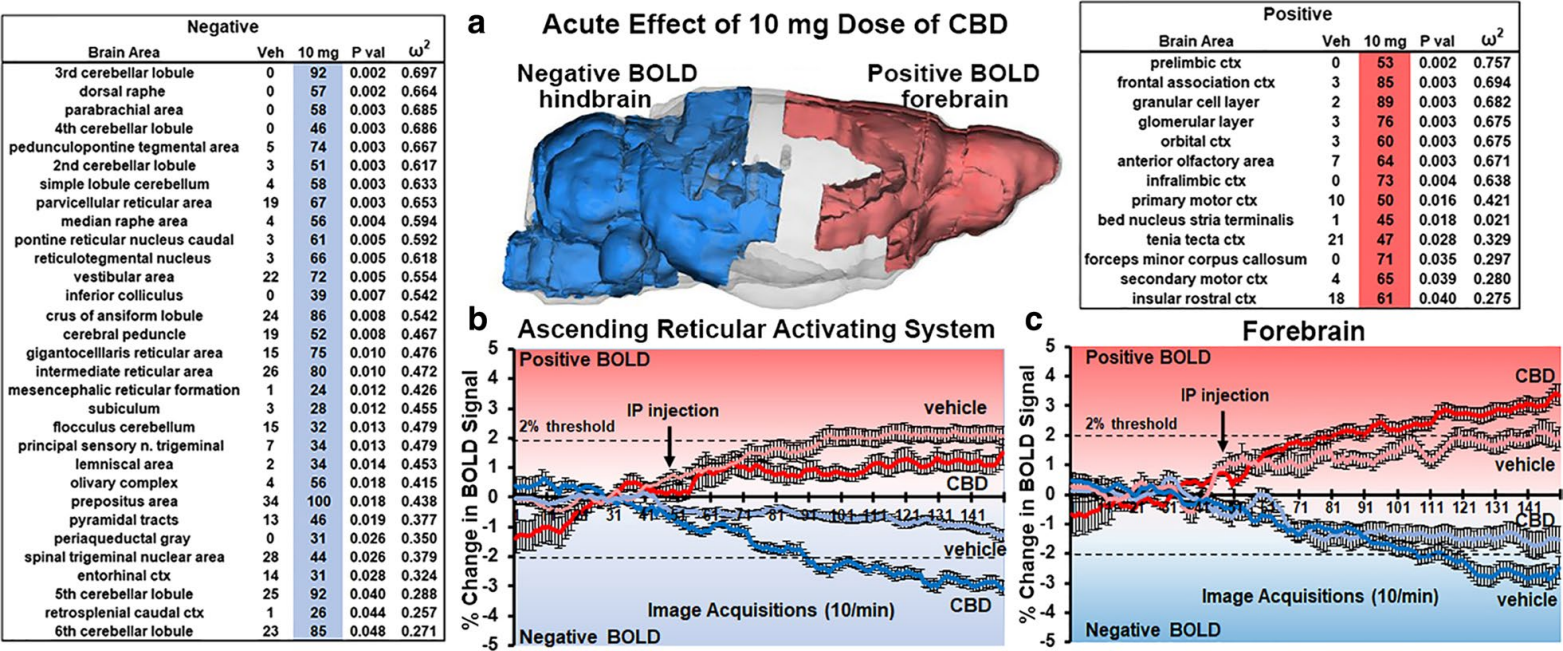
Acute Effects of 10 mg Dose of CBD. The tables show a truncated list of 31 and 13 out of 138 brain areas ranked in order of their significance for changes in negative and positive BOLD volume of activation, respectively in response to the 10 mg/kg I.P. dose of CBD. The 3D image (**a**) summarizes the location of these brain areas presenting with positive (red) and negative (blue) BOLD volume of activation. Below are graphs of BOLD signal change over the 15 min imaging session for the ascending reticular activating system (ARAS) **(b**) and forebrain (**c**). Comparisons are made between vehicle and the 10 mg dose of CBD. Shades of red denote positive changes and shades of blue negative changes. For ARAS positive BOLD: (p = 0.0045; CBD < Veh). For ARAS negative BOLD: (p < 0.0001; CBD > Veh). For forebrain positive BOLD (p = 0.0199; CBD > Veh). For forebrain negative BOLD: (p = 0.026 CBD > Veh)

Figure 2 summarizes the effect of the 10 mg/kg of CBD on BOLD signal**,** with tables showing significant changes, negative and positive, BOLD activation. The 3D image summarizes the location of the brain areas presenting with positive (red) and negative (blue) activation. The distribution is polarized along the rostral-caudal axis with positive BOLD localized to the forebrain and negative signal changes confined to the hindbrain. The forebrain areas are represented by the olfactory system (e.g., granular and glomerular layers of olfactory bulb, anterior olfactory area, and tenia tecta) and the prefrontal cortex (e.g., prelimbic, frontal association, orbital, infralimbic, 2nd and primary cortices). Connecting these bilateral forebrain areas is the forceps minor of the corpus callosum. The negative BOLD in the hindbrain is represented by the cerebellum (e.g., 2nd–6th lobules, simple lobule, crus of ansiform lobule flocculus) and ascending reticular activating system (ARAS) (e.g., dorsal raphe, parabrachial nucleus (n.), parvicellular reticular n., gigantocellularis, pedunculopontine n., pontine reticular n. mesencephalic reticular n.). In addition to volume of activation i.e. number of voxels activated with CBD treatment, changes in positive and negative BOLD signal over time, another measure of functional activity, are presented for the ARAS and forebrain. Each time point or image acquisition is the average BOLD signal of all brain areas comprising the ARAS and all areas in the forebrain. CBD has no significant effect on positive BOLD signal in the ARAS; instead, vehicle causes a greater, albeit small and at the level of threshold (above noise) increase in CBD (2-way ANOVA, F_(1,88)_ = 8.48, p = 0.0045; CBD < Veh). In the forebrain this pattern was reversed. CBD caused a significant increase in positive BOLD versus vehicle (F_(1,167)_ = 6.025, p = 0.0199; CBD > Veh) while being significantly less than vehicle for negative BOLD (F_(1,167)_ = 5.008, p = 0.026 CBD < Veh).

**Fig. 3:**
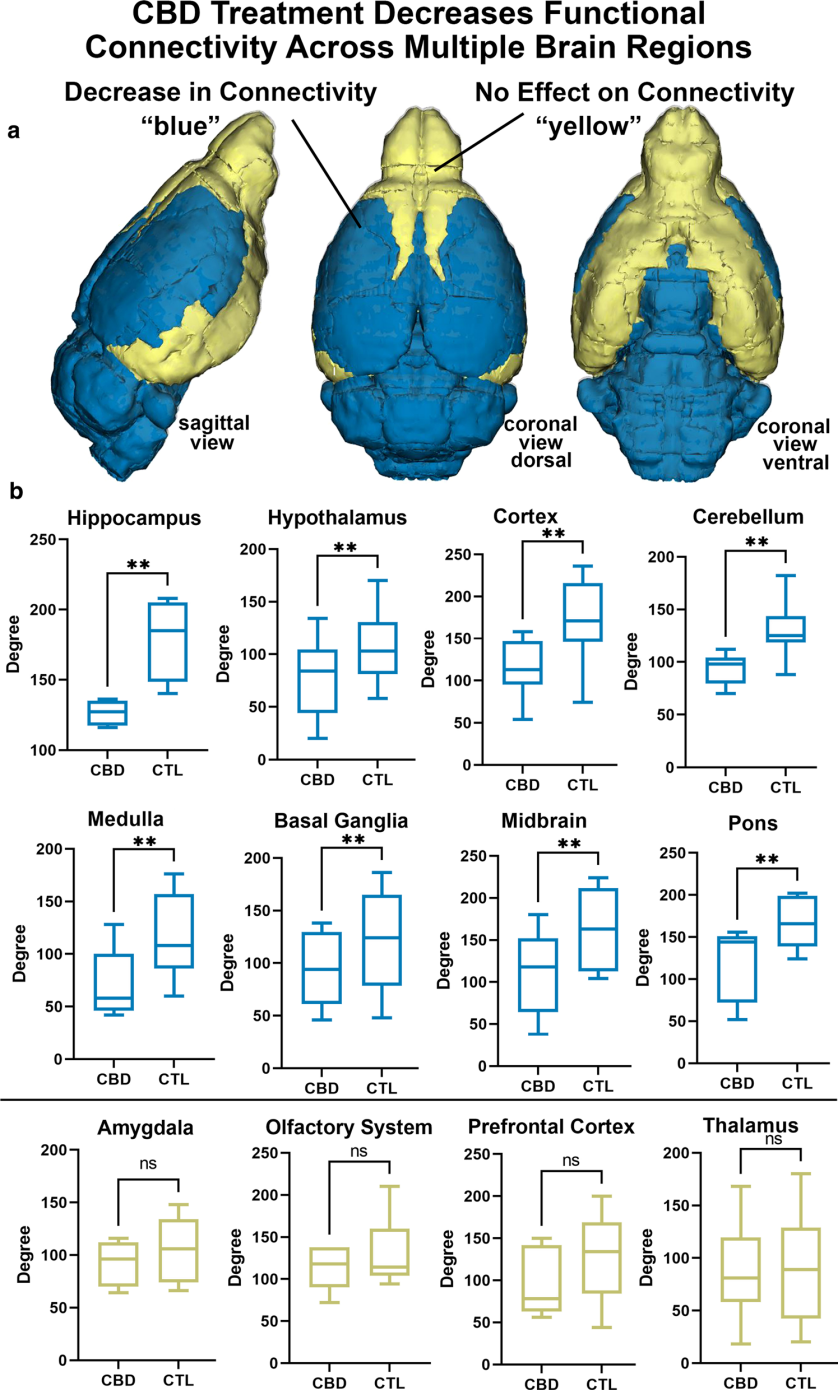
Regional Changes in Connectivity. Shown are 3D color coded images summarizing CBD-induced changes in connectivity (**a**) and box and whiskers plots (**b**) depicting differences in degree centrality in various subregions between vehicle and rats treated with 10 mg/kg CBD 60 min prior to imaging. The CBD group had significantly lower degree centrality of nodes within the hippocampus, hypothalamus, cortex, cerebellum, brainstem, basal ganglia, midbrain, and pons (**p < 0.05, not significant = ns). There were no significant differences in degree within nodes of the amygdala, olfactory system, prefrontal cortex, or thalamus

**Fig. 4:**
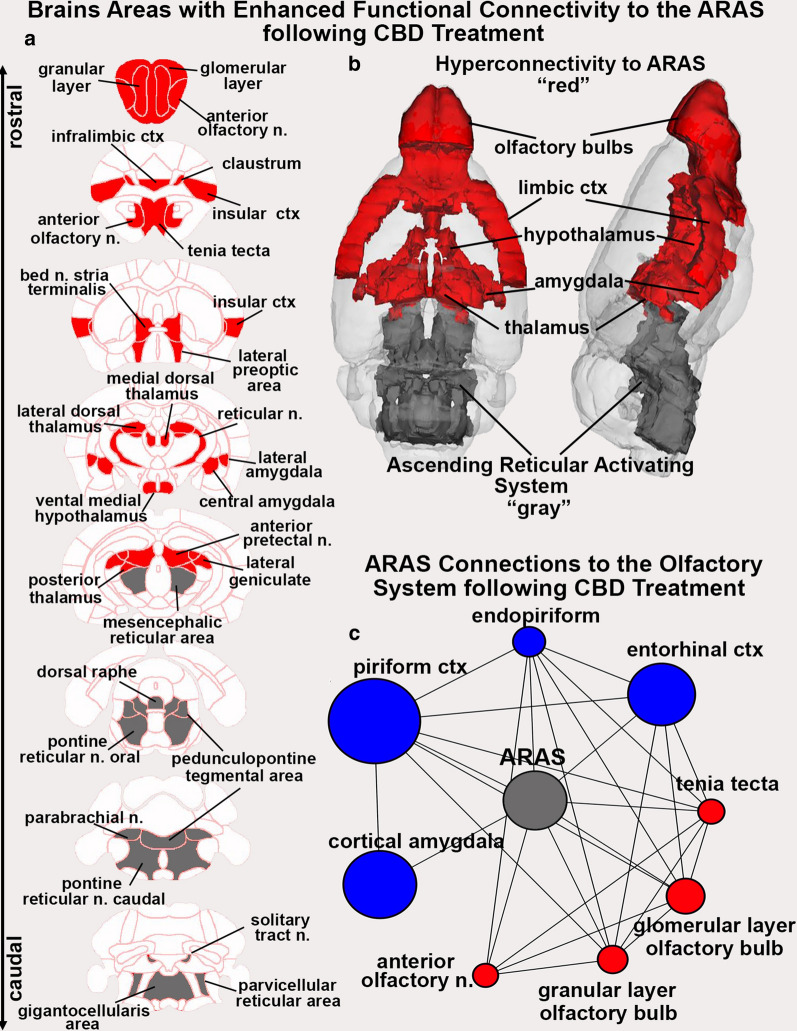
Hyperconnectivity to the ARAS with CBD Treatment. Shown to the left (**a**) are 2D axial maps showing the location of brain areas (red) with enhanced coupling to the ARAS following CBD treatment. Areas in gray denote the location of brain areas comprising the ARAS. The 2D images are summarized in the 3D reconstruction of the red and gray brain areas (**b**). The circle of connections beneath (**c**), display the neighboring nodes of the ARAS in the CBD treated group within the olfactory system. Nodes that have a greater degree centrality in the CBD group have been colored red, while nodes that have a greater degree centrality in the vehicle group have been colored blue. Node size has been scaled to reflect the relative difference in degree centrality between the vehicle and CBD group, with the larger nodes reflecting a larger difference in degree centrality

Looking at changes in connectivity (degrees) between CBD and controls in major brain areas reveals a pattern of functional coupling consistent with the pronounced negative BOLD observed with phMRI (Fig. 3). Mice treated with 10 mg/kg CBD prior to imaging showed a significant decrease in coupling in all the hindbrain regions, midbrain, hypothalamus, and cortex. While there were no significant differences seen in forebrain regions of olfactory system, prefrontal cortex, the thalamus, or the amygdala. Interestingly, when the ARAS is combined as a node, the same pattern of uncoupling appears between the ARAS and all these major brain regions, except for the olfactory system, prefrontal cortex, thalamus, and amygdala (Additional file 1: Figure S4). The ARAS as a node, under the influence of CBD, may not be significantly less than these brain regions but it is positively correlated with specific brain areas within these areas (Fig. 4). The 2D maps show the neuroanatomical position of brain areas with increased coupling to the ARAS (highlighted in red) following CBD treatment compared to vehicle. The areas shown in gray comprise the ARAS. A 3D reconstruction of these areas is shown to the left. The olfactory system is a large part of the hyperconnectivity between the ARAS and these limbic forebrain regions. The wire diagram below shows the significant negative (blue) and positive (red) connections between the ARAS and specific areas in the primary olfactory system.

**Fig. 5:**
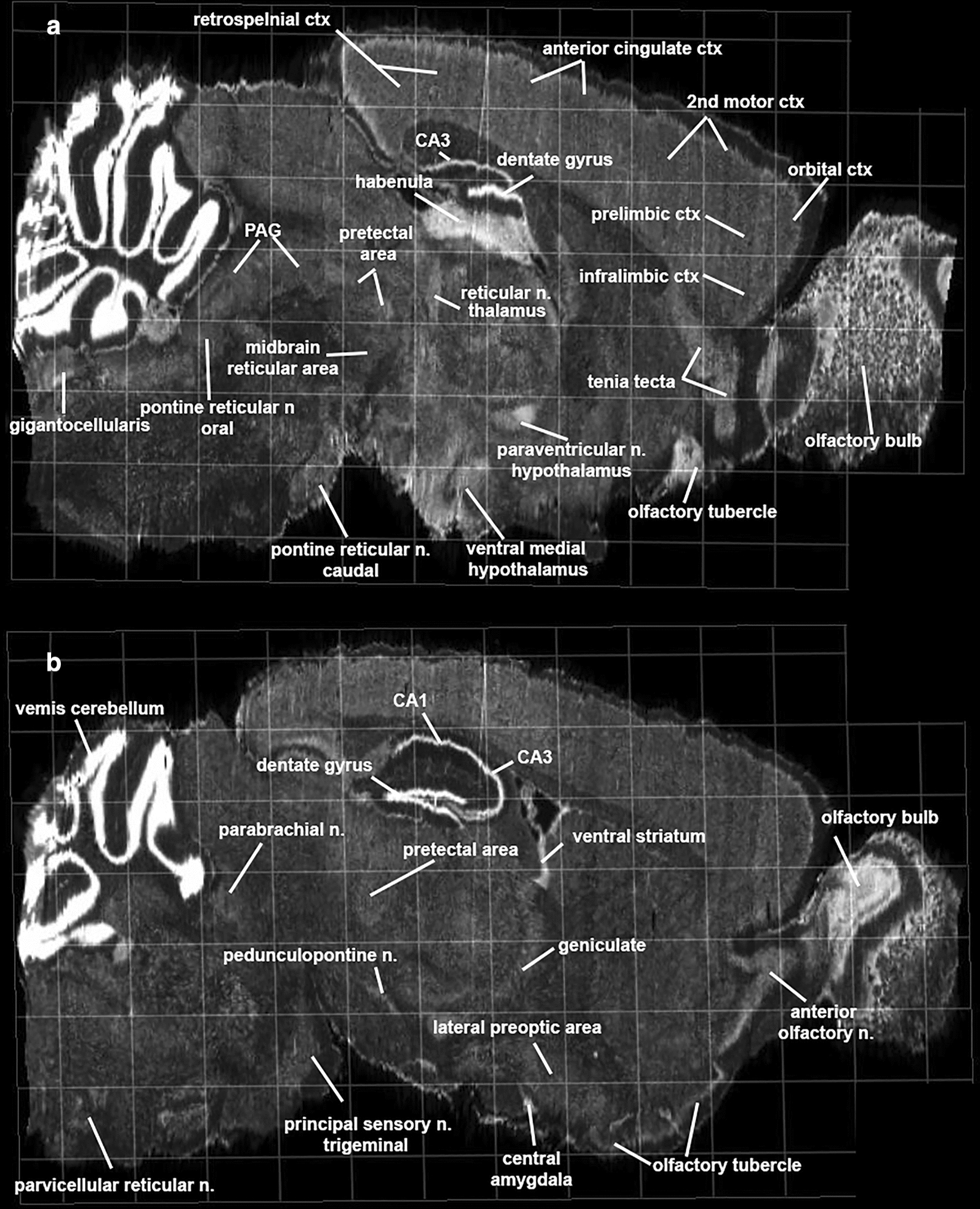
*N*-acyl-phosphatidylethanolamines -specific phospholipase D mRNA. Shown are autoradiograms of in situ hybridization of NAPD-PLD messenger RNA in mouse brain. The sagittal section A and B extend medial to lateral. Abbreviations PAG—periaqueductal gray. Image credit: Allen Institute

Shown in Fig. 5 are autoradiograms of in situ hybridization of NAPE-PLD from the Allen Brain Atlas [32]. These are sagittal sections that extend from the midline laterally from top (A.) to bottom (B.). The signal intensity reflects the level of mRNA in specific brain areas. Note, in general, the forebrain olfactory system and hindbrain cerebellum have a high density of NAPE-PLD mRNA. All the areas comprising the ARAS have NAPE-PLD mRNA (A. gigantocellularis, pontine reticular n, oral, midbrain reticular n., pontine reticular n. caudal; B. parabrachial n., pedunculopontine n., parvicellular reticular n.). Many of the areas shown in the 2D maps of positive ARAS connectivity to thalamus, amygdala, prefrontal, and olfactory system show high levels of NAPE-PLD mRNA (e.g., olfactory bulb, anterior olfactory n., tenia tecta, infralimbic ctx, lateral preoptic area, ventral medial hypothalamus, central amygdala, geniculate, reticular n. pretectal n.).

## Discussion

CBD produced activation in the prefrontal cortex and deactivation in the brainstem/cerebellum, particularly within the ascending reticular activating system (ARAS), with no changes apparent in the hypothalamus, amygdala, basal ganglia, or hippocampus. rsFC showed a decoupling of the hindbrain and midbrain regions, particularly the ARAS following CBD treatment. The integrated activity of the ARAS affects all aspects of cognitive and emotional behavior [33]. Interestingly, there were several areas of the brain that were positively coupled with the ARAS following CBD treatment. These area colocalize with a high density of *N*-acyl-phosphatidylethanolamines (NAPE) by a NAPE-specific phospholipase D (NAPE-PLD) mRNA [32]. NAPE-PLD is a constitutively active enzyme involved in the biosynthesis of *N*-acylethanolamines, signaling lipids molecules like anandamide [34]. The rsFC showed hyperconnectivity and hypoconnectivity that was consistent with the phMRI data. These findings are discussed with respect to the many studies showing CBD can affect the emotional and cognitive behavior associated with anxious and fearful events and NAPE-PLD as a putative mechanism of action.

### Human imaging and CBD

Several studies have used imaging to characterize the acute effect of CBD on brain activity in humans. SPECT imaging in volunteers diagnosed with anxiety disorder shows CBD increases blood flow in the cingulate cortex and reduces flow in the hippocampus while decreasing anxiety [35]. BOLD imaging in healthy volunteers, shows CBD decreases activation in the cerebellum, anterior cingulate, and amygdala, in a visual fear paradigm but not to neutral stimuli [15]. In healthy volunteers, CBD enhances caudate and hippocampal activation and fronto-striatal connectivity during salience processing [12, 13] and under resting state conditions [16], enhances auditory and visual processing [19] and effects working and episodic memory associated with an increase in blood flow to the hippocampus [14]. CBD alters functional coupling in cerebellum, frontal, and occipital cortices in patients with treatment resistant epilepsy [17] and attenuates hippocampal-striatal functional connectivity in psychosis patients [18]. All these studies gave oral doses of CBD prior to scanning, thus establishing a baseline of resting state blood flow that changed with different task-related paradigms or differed from placebo or healthy controls in response to a preexisting condition.

### Polarized positive and negative BOLD

The data reported here in awake mice are not easily compared to the human imaging studies. As the only study of its kind, we are looking at the immediate, dose-dependent effects of CBD, administered I.P., on brain activity across 138 different brain areas. The dose response showed the same inverted U-shape reported in many behavioral studies following systemic injection of CBD in rodents [24, 25, 36, 37] and humans [38]. The 10 mg/kg I.P. dose stood out as being the most effective, corroborating the many studies in rodents employing this dose [24–26, 36]. Within 10 min of injection, there was increase in positive BOLD signal in the prefrontal cortex/olfactory system and negative BOLD signal in the brainstem/cerebellum, particularly in brains areas comprising the ARAS. The absence of BOLD signal change in brain areas between the rostral/caudal axis of the brain (e.g., hippocampus, sensorimotor cortices, thalamus, hypothalamus, amygdala, and basal ganglia) made this pattern of activation and deactivation especially intriguing. This is unlike anything reported in awake animal imaging following tests on numerous CNS active drugs [8–11, 39–47]. Here we show the positive and negative changes in BOLD signal occur within 10 min of injection, and while CBD is known to rapidly penetrate the brain within seconds following systemic administration [48], its effects could be orchestrated easily by both peripheral and central targets. It should be noted that a pharmacokinetics study by Holzek et al. reported a much slower time course for brain penetrance following systemic CBD treatment [49].

### CBD targets

The primary targets for systemic CBD are unknown. Possible candidates include the cannabinoid CB1 receptors [50, 51], serotonin 5HT1a receptor, and the transient receptor potential vanilloid type 1 (TRPV1) [7, 52]. One important consequence of systemic CBD is the dramatic change in the CNS lipidome including increases in anandamide and related lipids that occurred in a NAPE-PLD dependent manner [22]. Do the CBD induced site-specific change in brain activity reported in our study match the distribution the putative targets noted above? CB1 receptors are localized to olfactory system, hippocampus, basal ganglia, cerebellum, and neocortex but very little in brainstem [53]. High densities of 5HT1a receptors are localized to prefrontal cortex, amygdala, hippocampus, and hypothalamus, while receptors are undetectable in the cerebellum and marginal in the brainstem [54, 55]. TRPV1 is expressed throughout the CNS with the highest density of receptors localized to the hippocampus, amygdala, hypothalamus, prefrontal cortex, and cerebellar cortex, while the lowest levels are in the brainstem [56–58]. NAPE-PLD distribution as shown in Fig. 5 is highest in hippocampus, cerebellum, olfactory system, and site-specific areas of the thalamus, amygdala, and brainstem [59, 60]. The distribution of NAPE-PLD seems to fit the activity pattern of CBD, specifically with respect to the ARAS. However, neither the distribution of CB1, 5HT1a, TRPV1, nor NAPE-PLD alone or together, can explain the absence of responsiveness of large parts of the brain to CBD or the polarization of BOLD signal. CBD has a complex pharmacology with activity at multiple targets beyond those discussed above (review see [7]). Given the promiscuity of CBD, there is no obvious explanation for the pattern of BOLD signal change based on location of a single target in the brain.

### Autonomic arousal and stress

The negative BOLD in brain areas that comprise the ARAS would suggest a decrease in brain activity and a reduction in autonomic arousal. Acute and chronic dosing of CBD in humans and animals has no appreciable effect on blood pressure, heart rate or blood flow [61]. However, CBD mediates the emotional and cardiovascular response to stress. CBD blunts the increased heart rate and blood pressure associated with the stress of forced immobilization [26, 62, 63] and increase in blood pressure, heart rate and immobility behavior in response to fear associated with the memory of an aversive condition [64]. CBD reduces immobility and escape behavior in mice exposed to a wild snake [65], altering the innate fear and aversion to predation. Rats exposed to cats present with long-lasting anxiogenic behavior that can be reduced with CBD [66]. Thus, CBD can reduce the anxiety, fear and immobilization associated with stressful or life-threatening events.

### Speculation

Interesting by its very nature, awake fMRI is a model of restraint stress, requiring the immobilization of the head to minimize artifacts. Acclimation is used to reduce the autonomic measures of stress [28], meaning the test subjects have a history of stress and adaptation. In additional, there is probably some emotional/physical stress associated with drug delivery during testing. The deactivation of the ARAS as interpreted by the increase in negative BOLD would be anticipated under these conditions and provide a neural target for CBD that would explain the reduction in autonomic and behavioral responses associated with anxious and potentially harmful environmental stimuli. Is the negative BOLD response to the ARAS unique to the acclimation process, i.e., is it an adaptation that has primed the lipidome to function under a new set of environmental pressures?

### Evolutionary significance

Is there a neurobiological explanation in the evolution of animals that would favor the global pattern of deactivation and uncoupling of functional circuits observed in much of the brain while favoring activation and hyperconnectivity to the forebrain and olfactory system by the ARAS? CDB is most effective when given to patients or animals presenting with high anxiety and fear. Specifically, it can reduce heart rate and blood pressure during heighten sympathetic arousal but has little to no intrinsic effect on these autonomic measures under homeostatic conditions. CBD is acting on reactive neural circuitry—the brain’s prewired, immediate response to threat. Freezing or behavioral arrest is a natural response to predator threat as a way of reducing detection. However, when the interaction becomes physical, the behavioral arrest can escalate into tonic immobility, an innate response of extreme physical inactivity [67]. This last chance to escape predation is commonly referred to as death feigning [68]. The immobility arises from descending neurons in the medullary, pontine reticular formation that suppress spinal motor neuron activity [69]. The neural circuitry of tonic immobility includes much of the ARAS described here [70–72], in addition to the PAG [73, 74], basolateral and central amygdala [75], and medial dorsal thalamus [76]. Treatment with CBD affects the BOLD signal and rsFC in all these areas. One of the more fascinating aspects of tonic immobility is continued sensory perception, i.e., animals feigning death can process sensory information and are aware of their environment [72, 77, 78]. The activation of the olfactory system by CBD would allow animals to continually survey their environment for the presence of the predator. The hypothesis that CBD could be affecting endocannabinoid signaling through NAPE-PLD is purely speculative but given the unique pattern of global brain activity caused by CBD treatment may warrant investigation and has far reaching implications. The neuropsychiatric trauma associated with life threatening experiences, e.g. PTSD, may crystalize around this phylogenetically old neural circuitry primal to survival [79]. Indeed, the evidence for CBD and endocannabinoid signaling playing a significant role in emotional regulation in neuropsychiatric disorders is growing [80].

### Limitations

(1) These studies did not address sex difference in CBD responsivity. The previous study investigating CBD’s effects on the brain lipidome were in female mice [22], so there is evidence that CBD has a significant effect on the brain within the time period; however, future studies will need to address if the changes in brain connectivity shown here in male mice are also measured in female mice. (2) Resting state functional connectivity was collected while rat were lightly anesthetized with isoflurane to minimize motion and physiological stress during “resting state” BOLD functional connectivity imaging (review see [81]). Anesthesia may reduce the magnitude of the BOLD signal but does not disrupt the connectivity as demonstrated across species and under different physiological conditions [82–86]. In this study the rsFC showed hyperconnectivity and hypoconnectivity that was consistent with the phMRI data. (3) There were no measures of NAPE-PLD activity in response to CBD challenge. These studies were not originally designed to test the involvement of NAPE-PLD.

### Summary

phMRI in awake mice was used to assess the immediate dose-dependent effects of CBD on global brain activity. The pattern of brain activity was unique and unexpected, characterized by activation in the prefrontal cortex and deactivation in the brainstem/cerebellum, particularly in the ARAS. These data provide a novel framework to understand how CBD drives CNS changes that can be targeted for therapeutics. The putative target and mechanism of action is NAPE-PLD the enzyme responsible for the biosynthesis of lipid signaling molecules like anandamide.

## Supplementary Information

**Additional file 1.** Supplementary Data.

## Data Availability

All data can be accessed through a link to Mendeley. DOI to follow.
