# Supplementary material for: Cannabidiol has a unique effect on global brain activity: a pharmacological, functional MRI study in awake mice
Source: J Transl Med. 2021 May 24;19:220. doi: 10.1186/s12967-021-02891-6 (PMC8142641; doi:10.1186/s12967-021-02891-6)
Supplement: Supplementary file 1 — Additional file 1. Supplementary Data. [file 12967_2021_2891_MOESM1_ESM.docx]

**Additional Figure S1.** Motion Artifact


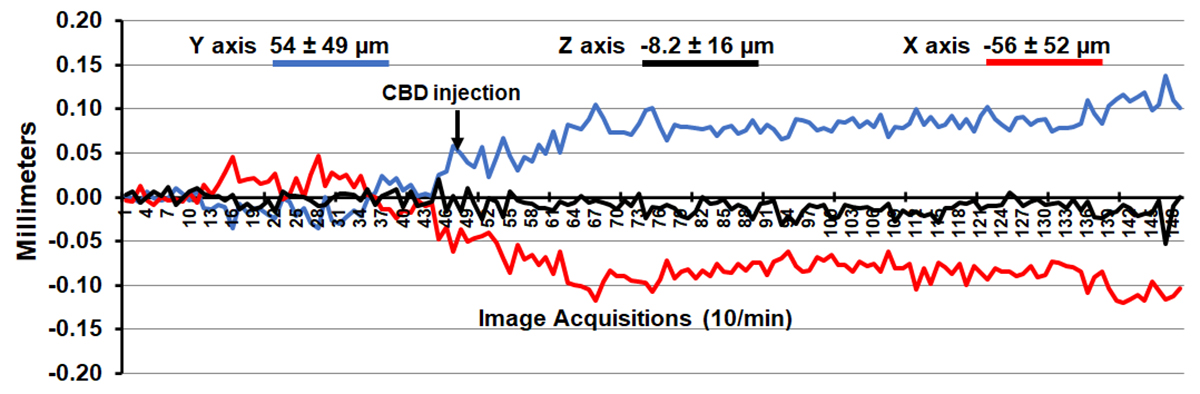
Shown is the degree of motion artifact recorded over the 15 min imaging protocol. The data are reported as the mean and standard deviation in micrometers for axis Y, Z and X for the 18 mice injected with CBD. The greatest movement was in the X and Y axis (left to right, up and down, respectively) and occurred within the first two min of injection (acquisitions 50-70) but stabilized over the remaining imaging session.

**Additional Figure S2.** Neuroanatomical fidelity

Shown are representative examples of brain images collected during a single imaging session using a multi-slice spin echo, RARE (rapid acquisition with relaxation enhancement) pulse sequence. The row on the top shows axial sections collected during the anatomical scan taken at the beginning of each imaging session using a data matrix of 128 × 128, 18 slices in a field of view of 1.8 cm. The row below shows the same images but collected for functional analysis using HASTE, a RARE pulse sequence modified for faster acquisition time. These images were acquired using the same field of view and slice anatomy but a larger data matrix of 96 × 96. Note the anatomical fidelity between the functional images and their original anatomical image. The absence of any distortion is necessary when registering the data to atlas to resolve 136 segmented brain areas.


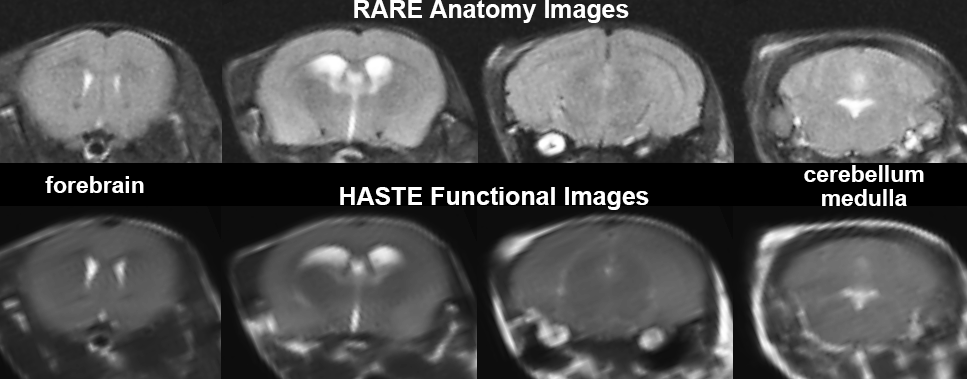


**Additional Figure S3 CBD Dose Response**

Shown are the mean (± SD) plasma levels of CBD (ng/ml) for each of the three doses. Samples were collected within 60 min of the initial injection following the imaging session. There are four samples per dose. B) shows the plasma levels of THC discovered in the same doses of CBD. The levels of CBD and THC were significantly different between doses (One way ANOVA F_(2,9)_ = 89.69, and 24.90 respectively, p<.0001). Tukey’s multiple comparisons showed a significant difference between the 3 and 30 mg doses and the 10 and 30 mg doses for both CBD and THC measures. *** p<0.001, ****p<0.0001.

**
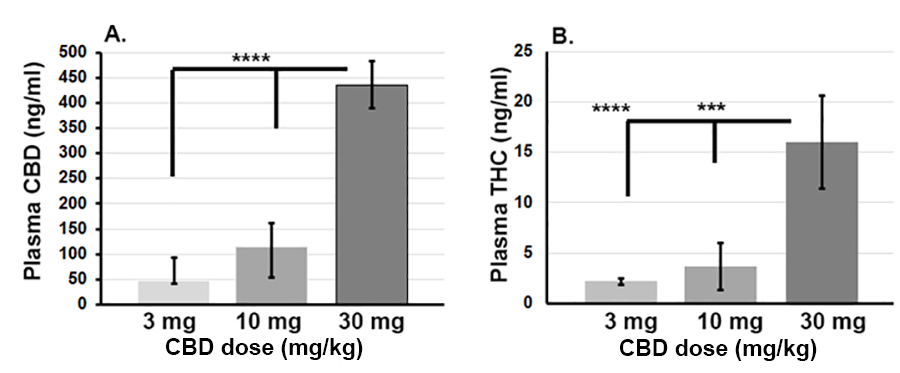
**

**Additional Figure S4** ARAS Connectivity

**
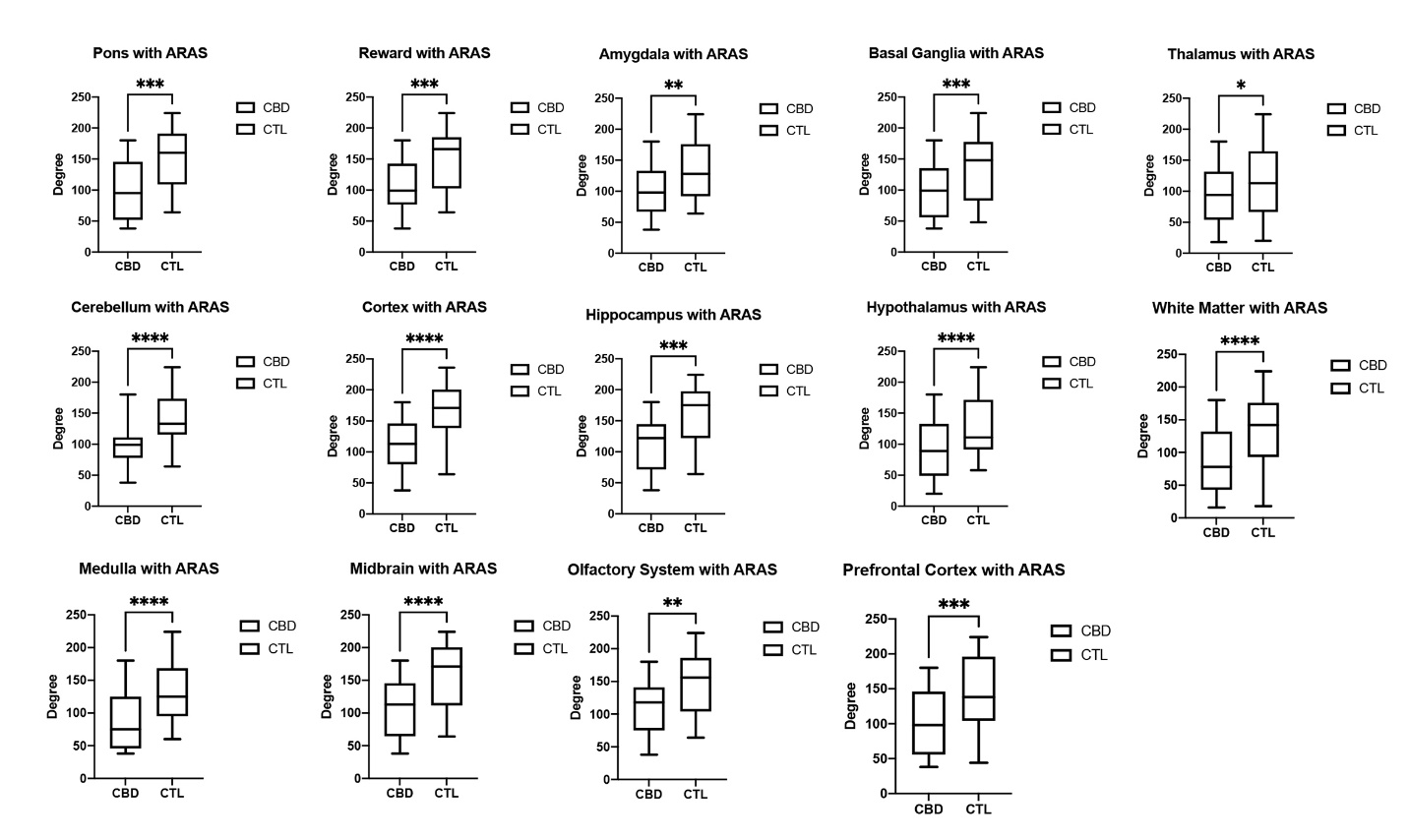
**Shown are box and whiskers plots depicting differences in degree centrality in various subregions between vehicle (CTL) and rats treated with 10mg/kg CBD 60 min prior to imaging.

**Additional Table S1**

| **Positive BOLD** | | | | | | |
| --- | --- | --- | --- | --- | --- | --- |
| **Brain Area** | **Veh** | **3mg** | **10mg** | **30mg** | **P val** | **ω^2^** |
| **glomerular layer** | **3** | **9** | **76** | **34** | **0.001** | **0.806** |
| **granular cell layer** | **2** | **8** | **89** | **23** | **0.001** | **0.757** |
| **anterior olfactory area** | **7** | **19** | **64** | **33** | **0.001** | **0.701** |
| **flocculus cerebellum** | **29** | **41** | **10** | **19** | **0.001** | **0.635** |
| **medullary reticular ventral area** | **0** | **27** | **5** | **0** | **0.001** | **0.578** |
| **reticulotegmental nucleus** | **25** | **0** | **0** | **0** | **0.002** | **0.546** |
| **locus coeruleus** | **0** | **25** | **0** | **0** | **0.002** | **0.537** |
| **frontal association ctx** | **3** | **12** | **85** | **26** | **0.003** | **0.482** |
| **crus of ansiform lobule** | **21** | **40** | **3** | **14** | **0.005** | **0.448** |
| **lateral lemniscus** | **42** | **33** | **8** | **4** | **0.006** | **0.428** |
| **orbital ctx** | **3** | **8** | **60** | **8** | **0.007** | **0.411** |
| **pontine area** | **29** | **15** | **3** | **27** | **0.007** | **0.401** |
| **spinal trigeminal nuclear area** | **22** | **33** | **9** | **38** | **0.007** | **0.4** |
| **infralimbic ctx** | **0** | **0** | **73** | **56** | **0.008** | **0.386** |
| **prelimbic ctx** | **0** | **5** | **53** | **2** | **0.009** | **0.384** |
| **cuneate area** | **50** | **48** | **0** | **0** | **0.009** | **0.381** |
| **5th cerebellar lobule** | **0** | **7** | **0** | **0** | **0.01** | **0.368** |
| **paramedian lobule** | **10** | **33** | **0** | **8** | **0.01** | **0.368** |
| **6th cerebellar lobule** | **17** | **38** | **0** | **3** | **0.011** | **0.364** |
| **pontine reticular nucleus caudal** | **26** | **13** | **0** | **0** | **0.011** | **0.361** |
| **solitary tract area** | **5** | **37** | **0** | **21** | **0.016** | **0.322** |
| **parvicellular reticular area** | **31** | **30** | **4** | **27** | **0.017** | **0.318** |
| **vestibular area** | **3** | **42** | **0** | **11** | **0.017** | **0.318** |
| **simple lobule cerebellum** | **12** | **26** | **0** | **0** | **0.021** | **0.299** |
| **4th cerebellar lobule** | **12** | **10** | **0** | **0** | **0.022** | **0.294** |
| **principal sensory n. trigeminal** | **25** | **28** | **8** | **10** | **0.023** | **0.291** |
| **lemniscal area** | **15** | **16** | **0** | **0** | **0.024** | **0.286** |
| **entorhinal ctx** | **30** | **40** | **18** | **16** | **0.028** | **0.27** |
| **prepositus area** | **0** | **9** | **0** | **14** | **0.031** | **0.26** |
| **3rd cerebellar lobule** | **7** | **9** | **0** | **0** | **0.038** | **0.239** |
| **primary motor ctx** | **10** | **14** | **50** | **19** | **0.04** | **0.235** |
| **inferior colliculus** | **20** | **39** | **3** | **5** | **0.04** | **0.233** |
| **10th cerebellar lobule** | **0** | **0** | **0** | **3** | **0.042** | **0.23** |
| **periaqueductal gray** | **19** | **11** | **1** | **12** | **0.046** | **0.22** |
| **caudate putamen** | **6** | **10** | **48** | **27** | **0.049** | **0.214** |
| **visual 1 ctx** | **17** | **32** | **5** | **14** | **0.051** | **0.21** |
| **gigantocellularis reticular area** | **31** | **23** | **1** | **26** | **0.052** | **0.208** |
| **subiculum** | **18** | **22** | **5** | **6** | **0.066** | **0.185** |
| **stria medullaris** | **0** | **0** | **0** | **33** | **0.068** | **0.182** |
| **cerebellar nuclear area** | **0** | **9** | **0** | **0** | **0.074** | **0.173** |
| **intermediate reticular area** | **23** | **28** | **3** | **28** | **0.075** | **0.171** |
| **forceps minor corpus callosum** | **0** | **0** | **71** | **22** | **0.077** | **0.002** |
| **tenia tecta ctx** | **21** | **19** | **47** | **29** | **0.077** | **0.169** |
| **olivary complex** | **30** | **37** | **5** | **4** | **0.079** | **0.167** |
| **globus pallidus** | **1** | **15** | **37** | **34** | **0.087** | **0.157** |
| **pontine reticular nucleus oral** | **12** | **11** | **2** | **3** | **0.088** | **0.156** |
| **accumbens core** | **6** | **4** | **56** | **19** | **0.095** | **0.148** |
| **parabrachial area** | **0** | **17** | **0** | **0** | **0.105** | **0.138** |
| **facial nucleus** | **55** | **23** | **0** | **25** | **0.106** | **0.137** |
| **secondary motor ctx** | **4** | **13** | **65** | **9** | **0.107** | **0.136** |
| **2nd cerebellar lobule** | **20** | **11** | **0** | **0** | **0.108** | **0.135** |
| **insular rostral ctx** | **18** | **25** | **61** | **16** | **0.108** | **0.135** |
| **pedunculopontine tegmental area** | **7** | **12** | **0** | **13** | **0.108** | **0.135** |
| **bed nucleus stria terminalis** | **1** | **4** | **45** | **9** | **0.126** | **0.12** |
| **fornix** | **0** | **13** | **0** | **0** | **0.129** | **0.117** |
| **fimbria hippocampus** | **8** | **29** | **28** | **14** | **0.131** | **0.006** |
| **external capsule** | **12** | **6** | **26** | **29** | **0.136** | **0.053** |
| **central medial thalamic area** | **0** | **0** | **22** | **10** | **0.137** | **0.111** |
| **lateral septal area** | **6** | **9** | **52** | **22** | **0.143** | **0.081** |
| **temporal ctx** | **18** | **13** | **0** | **13** | **0.156** | **0.097** |
| **optic tract** | **7** | **36** | **13** | **40** | **0.166** | **0.091** |
| **stria terminalis** | **0** | **0** | **0** | **0** | **0.167** | **0.091** |
| **retrosplenial caudal ctx** | **24** | **27** | **0** | **4** | **0.167** | **0.09** |
| **reuniens thalamic area** | **2** | **23** | **17** | **0** | **0.18** | **0.103** |
| **caudal piriform ctx** | **35** | **26** | **25** | **42** | **0.185** | **0.08** |
| **posterior hypothalamic area** | **0** | **42** | **33** | **0** | **0.192** | **0.076** |
| **anterior cingulate area** | **2** | **10** | **51** | **28** | **0.201** | **0.071** |
| **posterior thalamic area** | **0** | **0** | **0** | **17** | **0.213** | **0.065** |
| **basal amygdaloid area** | **18** | **30** | **0** | **35** | **0.216** | **0.064** |
| **dorsal hippocampal commissure** | **22** | **14** | **0** | **0** | **0.218** | **0.087** |
| **lateral geniculate** | **15** | **0** | **17** | **18** | **0.225** | **0.06** |
| **anterior hypothalamic area** | **41** | **33** | **25** | **22** | **0.228** | **0.058** |
| **accumbens shell** | **10** | **27** | **81** | **25** | **0.241** | **0.053** |
| **internal capsule** | **5** | **15** | **36** | **30** | **0.248** | **0.049** |
| **paraventricular hypothalamic area** | **0** | **13** | **0** | **0** | **0.254** | **0.002** |
| **anterior commissure** | **0** | **21** | **33** | **0** | **0.263** | **0.043** |
| **parietal ctx** | **0** | **10** | **8** | **8** | **0.289** | **0.104** |
| **dorsal raphe** | **15** | **0** | **0** | **0** | **0.306** | **0.027** |
| **lateral paragigantocellular area** | **45** | **43** | **33** | **31** | **0.311** | **0.015** |
| **primary somatosensory ctx** | **16** | **23** | **44** | **10** | **0.326** | **0.02** |
| **lateral dorsal thalamic area** | **0** | **17** | **5** | **19** | **0.339** | **0.016** |
| **paraventricular thalamic area** | **0** | **15** | **0** | **0** | **0.353** | **0.011** |
| **dentate gyrus** | **15** | **22** | **4** | **6** | **0.366** | **0.007** |
| **median raphe area** | **7** | **19** | **0** | **0** | **0.367** | **0.007** |
| **ventral medial hypothalamic area** | **17** | **39** | **15** | **9** | **0.377** | **0.004** |
| **mesencephalic reticular formation** | **16** | **16** | **8** | **8** | **0.384** | **0.002** |
| **central amygdaloid area** | **1** | **18** | **1** | **13** | **0.384** | **0.002** |
| **auditory ctx** | **21** | **19** | **2** | **12** | **0.421** | **0.008** |
| **7th cerebellar lobule** | **9** | **13** | **0** | **2** | **0.423** | **0.009** |
| **rostral piriform ctx** | **18** | **17** | **52** | **24** | **0.447** | **0.015** |
| **lateral reticular area** | **40** | **25** | **29** | **43** | **0.455** | **0.017** |
| **olfactory tubercles** | **26** | **30** | **52** | **30** | **0.458** | **0.018** |
| **retrosplenial rostral ctx** | **5** | **26** | **13** | **19** | **0.464** | **0.071** |
| **pituitary** | **28** | **36** | **21** | **15** | **0.477** | **0.022** |
| **interpeduncular area** | **26** | **30** | **0** | **0** | **0.505** | **0.029** |
| **superior colliculus** | **25** | **33** | **5** | **4** | **0.516** | **0.031** |
| **medial amygdaloid area** | **18** | **25** | **22** | **38** | **0.53** | **0.069** |
| **CA3** | **11** | **22** | **3** | **9** | **0.539** | **0.036** |
| **parafascicular thalamic area** | **0** | **0** | **0** | **50** | **0.546** | **0.037** |
| **anterior pretectal thalamic area** | **2** | **6** | **6** | **15** | **0.549** | **0.039** |
| **anterior thalamic area** | **3** | **11** | **7** | **3** | **0.592** | **0.048** |
| **lateral preoptic area** | **8** | **17** | **100** | **14** | **0.607** | **0.051** |
| **medial preoptic area** | **35** | **24** | **28** | **19** | **0.627** | **0.055** |
| **reticular thalamic area** | **3** | **6** | **41** | **29** | **0.628** | **0.055** |
| **substantia nigra** | **39** | **45** | **27** | **5** | **0.629** | **0.042** |
| **secondary somatosensory ctx** | **18** | **23** | **32** | **28** | **0.641** | **0.058** |
| **ambiguus area** | **43** | **18** | **20** | **43** | **0.647** | **0.059** |
| **ventral tegmental area** | **25** | **38** | **50** | **0** | **0.653** | **0.098** |
| **extended amygdala** | **9** | **13** | **50** | **9** | **0.659** | **0.15** |
| **medial septal area** | **12** | **0** | **0** | **0** | **0.663** | **0.031** |
| **corpus callosum** | **15** | **25** | **51** | **19** | **0.673** | **0.038** |
| **9th cerebellar lobule** | **7** | **18** | **3** | **15** | **0.693** | **0.067** |
| **ventral pallidum** | **30** | **25** | **45** | **45** | **0.717** | **0.072** |
| **lateral amygdaloid area** | **4** | **17** | **16** | **29** | **0.723** | **0.073** |
| **lateral caudal hypothalamic area** | **23** | **38** | **33** | **15** | **0.728** | **0.049** |
| **zona incerta** | **10** | **25** | **44** | **27** | **0.765** | **0.102** |
| **anterior amygdaloid area** | **27** | **22** | **40** | **15** | **0.771** | **0.082** |
| **lateral posterior thalamic area** | **0** | **6** | **0** | **8** | **0.819** | **0.09** |
| **8th cerebellar lobule** | **3** | **9** | **0** | **0** | **0.856** | **0.097** |
| **ventral thalamic area** | **7** | **3** | **32** | **15** | **0.858** | **0.014** |
| **endopiriform area** | **22** | **11** | **66** | **24** | **0.862** | **0.098** |
| **habenular area** | **0** | **8** | **0** | **0** | **0.872** | **0.048** |
| **diagonal band of Broca** | **23** | **23** | **32** | **27** | **0.88** | **0.101** |
| **cortical amygdaloid area** | **29** | **28** | **14** | **22** | **0.881** | **0.004** |
| **insular caudal ctx** | **23** | **25** | **33** | **21** | **0.881** | **0.102** |
| **lateral rostral hypothalamic area** | **20** | **32** | **31** | **20** | **0.883** | **0.102** |
| **CA1** | **12** | **12** | **8** | **10** | **0.905** | **0.083** |
| **dorsal medial hypothalamic area** | **10** | **27** | **22** | **29** | **0.908** | **0.025** |
| **medullary reticular dorsal area** | **21** | **25** | **15** | **11** | **0.94** | **0.113** |
| **medial mammillary area** | **26** | **33** | **30** | **44** | **0.974** | **0.121** |
| **medial dorsal thalamic area** | **6** | **5** | **13** | **5** | **0.983** | **0.016** |
| **medial geniculate** | **18** | **19** | **26** | **14** | **0.99** | **0.055** |

**Additional Table S2**

| **Negative BOLD** | | | | | | |
| --- | --- | --- | --- | --- | --- | --- |
| **Brain Area** | **Veh** | **3mg** | **10mg** | **30mg** | **P val** | **ω^2^** |
| **anterior olfactory area** | **44** | **46** | **4** | **0** | **0.001** | **0.691** |
| **glomerular layer** | **50** | **60** | **4** | **20** | **0.001** | **0.672** |
| **granular cell layer** | **39** | **61** | **0** | **7** | **0.001** | **0.653** |
| **frontal association ctx** | **64** | **39** | **2** | **2** | **0.001** | **0.555** |
| **lateral geniculate** | **0** | **22** | **9** | **0** | **0.002** | **0.448** |
| **2nd cerebellar lobule** | **3** | **16** | **51** | **45** | **0.002** | **0.464** |
| **reticulotegmental nucleus** | **3** | **17** | **66** | **0** | **0.003** | **0.519** |
| **parvicellular reticular area** | **19** | **11** | **67** | **32** | **0.003** | **0.477** |
| **ventral medial hypothalamic area** | **2** | **25** | **22** | **0** | **0.003** | **0.410** |
| **parabrachial area** | **0** | **10** | **58** | **0** | **0.003** | **0.440** |
| **rostral piriform ctx** | **24** | **27** | **6** | **7** | **0.004** | **0.387** |
| **orbital ctx** | **25** | **42** | **1** | **8** | **0.004** | **0.444** |
| **medullary reticular ventral area** | **30** | **7** | **58** | **0** | **0.005** | **0.456** |
| **simple lobule cerebellum** | **4** | **17** | **58** | **39** | **0.006** | **0.413** |
| **3rd cerebellar lobule** | **0** | **2** | **92** | **10** | **0.006** | **0.398** |
| **median raphe area** | **4** | **4** | **56** | **2** | **0.008** | **0.350** |
| **accumbens shell** | **11** | **17** | **0** | **2** | **0.009** | **0.330** |
| **flocculus cerebellum** | **15** | **24** | **32** | **6** | **0.010** | **0.409** |
| **6th cerebellar lobule** | **23** | **8** | **85** | **20** | **0.010** | **0.377** |
| **intermediate reticular area** | **26** | **28** | **80** | **46** | **0.011** | **0.336** |
| **mesencephalic reticular formation** | **1** | **14** | **24** | **3** | **0.011** | **0.312** |
| **7th cerebellar lobule** | **62** | **22** | **89** | **0** | **0.012** | **0.308** |
| **ventral pallidum** | **8** | **21** | **0** | **0** | **0.013** | **0.283** |
| **pontine reticular nucleus caudal** | **3** | **12** | **61** | **12** | **0.014** | **0.342** |
| **crus of ansiform lobule** | **24** | **16** | **86** | **37** | **0.014** | **0.351** |
| **spinal trigeminal nuclear area** | **28** | **19** | **44** | **15** | **0.015** | **0.337** |
| **gigantocelllaris reticular area** | **15** | **17** | **75** | **51** | **0.016** | **0.312** |
| **caudal piriform ctx** | **23** | **23** | **24** | **5** | **0.016** | **0.292** |
| **dorsal raphe** | **0** | **25** | **57** | **8** | **0.017** | **0.299** |
| **cuneate area** | **0** | **10** | **64** | **0** | **0.017** | **0.325** |
| **4th cerebellar lobule** | **0** | **1** | **46** | **15** | **0.018** | **0.311** |
| **pontine area** | **3** | **32** | **51** | **0** | **0.022** | **0.306** |
| **paramedian lobule** | **30** | **21** | **59** | **9** | **0.022** | **0.302** |
| **pedunculopontine tegmental area** | **5** | **10** | **74** | **8** | **0.023** | **0.276** |
| **pontine reticular nucleus oral** | **5** | **7** | **31** | **0** | **0.023** | **0.271** |
| **primary motor ctx** | **14** | **24** | **2** | **3** | **0.024** | **0.278** |
| **vestibular area** | **22** | **6** | **72** | **43** | **0.024** | **0.292** |
| **medullary reticular dorsal area** | **31** | **41** | **41** | **0** | **0.025** | **0.230** |
| **olfactory tubercles** | **25** | **27** | **7** | **2** | **0.026** | **0.236** |
| **insular rostral ctx** | **27** | **22** | **4** | **14** | **0.026** | **0.257** |
| **5th cerebellar lobule** | **25** | **2** | **92** | **87** | **0.028** | **0.301** |
| **lateral posterior thalamic area** | **0** | **26** | **3** | **0** | **0.029** | **0.205** |
| **olivary complex** | **4** | **28** | **56** | **8** | **0.031** | **0.245** |
| **diagonal band of Broca** | **13** | **36** | **0** | **0** | **0.033** | **0.192** |
| **prepositus area** | **34** | **3** | **100** | **64** | **0.034** | **0.276** |
| **primary somatosensory ctx** | **10** | **24** | **2** | **4** | **0.037** | **0.214** |
| **subiculum** | **3** | **15** | **28** | **11** | **0.041** | **0.226** |
| **inferior colliculus** | **0** | **5** | **39** | **25** | **0.042** | **0.228** |
| **periaqueductal gray** | **0** | **28** | **31** | **13** | **0.047** | **0.216** |
| **lemniscal area** | **2** | **6** | **34** | **0** | **0.049** | **0.225** |
| **lateral preoptic area** | **3** | **23** | **0** | **0** | **0.054** | **0.128** |
| **cerebellar nuclear area** | **30** | **10** | **60** | **71** | **0.056** | **0.217** |
| **8th cerebellar lobule** | **61** | **12** | **55** | **0** | **0.060** | **0.167** |
| **10th cerebellar lobule** | **37** | **14** | **75** | **55** | **0.060** | **0.242** |
| **visual 1 ctx** | **2** | **17** | **12** | **4** | **0.060** | **0.196** |
| **secondary motor ctx** | **20** | **19** | **1** | **2** | **0.063** | **0.182** |
| **anterior hypothalamic area** | **11** | **28** | **20** | **3** | **0.074** | **0.158** |
| **stria terminalis** | **0** | **0** | **0** | **0** | **0.074** | **0.072** |
| **solitary tract area** | **32** | **13** | **87** | **36** | **0.085** | **0.181** |
| **prelimbic ctx** | **29** | **14** | **0** | **10** | **0.087** | **0.189** |
| **anterior pretectal thalamic area** | **0** | **27** | **24** | **0** | **0.089** | **0.124** |
| **pituitary** | **9** | **35** | **19** | **0** | **0.094** | **0.118** |
| **principal sensory nucleus trigeminal** | **7** | **17** | **34** | **22** | **0.094** | **0.167** |
| **posterior thalamic area** | **0** | **3** | **3** | **0** | **0.099** | **0.111** |
| **reticular thalamic area** | **0** | **11** | **0** | **0** | **0.100** | **0.099** |
| **anterior commissure** | **11** | **21** | **0** | **0** | **0.102** | **0.138** |
| **retrosplenial caudal ctx** | **1** | **18** | **26** | **4** | **0.107** | **0.129** |
| **infralimbic ctx** | **0** | **17** | **0** | **0** | **0.108** | **0.163** |
| **medial mammillary area** | **11** | **27** | **24** | **0** | **0.115** | **0.083** |
| **9th cerebellar lobule** | **22** | **33** | **53** | **0** | **0.117** | **0.109** |
| **facial nucleus** | **8** | **30** | **50** | **11** | **0.124** | **0.124** |
| **auditory ctx** | **6** | **13** | **9** | **3** | **0.127** | **0.102** |
| **interpeduncular area** | **0** | **17** | **5** | **0** | **0.134** | **0.071** |
| **anterior amygdaloid area** | **14** | **18** | **0** | **0** | **0.134** | **0.066** |
| **pyramidal tracts** | **13** | **22** | **46** | **5** | **0.139** | **0.122** |
| **anterior cingulate area** | **7** | **8** | **0** | **13** | **0.140** | **0.103** |
| **entorhinal ctx** | **14** | **31** | **31** | **13** | **0.145** | **0.126** |
| **tenia tecta ctx** | **18** | **20** | **7** | **2** | **0.157** | **0.105** |
| **endopiriform area** | **11** | **25** | **0** | **4** | **0.167** | **0.061** |
| **secondary somaotsensory ctx** | **14** | **23** | **2** | **4** | **0.182** | **0.065** |
| **anterior thalamic area** | **6** | **20** | **4** | **5** | **0.187** | **0.066** |
| **central medial thalamic area** | **0** | **3** | **5** | **0** | **0.188** | **0.055** |
| **ambiguus area** | **28** | **15** | **33** | **0** | **0.217** | **0.041** |
| **lateral rostral hypothalamic area** | **14** | **20** | **0** | **2** | **0.224** | **0.036** |
| **medial preoptic area** | **4** | **35** | **0** | **0** | **0.247** | **0.030** |
| **superior colliculus** | **1** | **12** | **11** | **7** | **0.249** | **0.038** |
| **accumbens core** | **8** | **19** | **0** | **0** | **0.260** | **0.041** |
| **lateral dorsal thalamic area** | **3** | **26** | **0** | **0** | **0.261** | **0.032** |
| **central amygdaloid area** | **3** | **5** | **22** | **15** | **0.266** | **0.038** |
| **lateral lemniscus** | **2** | **18** | **29** | **7** | **0.269** | **0.084** |
| **temporal ctx** | **4** | **10** | **14** | **0** | **0.272** | **0.046** |
| **insular caudal ctx** | **21** | **17** | **10** | **7** | **0.286** | **0.018** |
| **posterior hypothalamic area** | **10** | **10** | **0** | **0** | **0.302** | **0.024** |
| **bed nucleus stria terminalis** | **5** | **12** | **0** | **0** | **0.304** | **0.033** |
| **lateral reticular area** | **23** | **9** | **28** | **0** | **0.311** | **0.012** |
| **paraventricular thalamic area** | **0** | **18** | **6** | **5** | **0.312** | **0.049** |
| **fornix** | **0** | **0** | **0** | **0** | **0.322** | **0.033** |
| **globus pallidus** | **3** | **9** | **0** | **5** | **0.324** | **0.035** |
| **lateral amygdaloid area** | **2** | **23** | **22** | **18** | **0.355** | **0.001** |
| **cortical amygdaloid area** | **25** | **27** | **28** | **12** | **0.361** | **-0.004** |
| **ventral thalamic area** | **0** | **5** | **2** | **1** | **0.365** | **-0.014** |
| **dentate gyrus** | **7** | **20** | **24** | **6** | **0.373** | **0.005** |
| **basal amygdaloid area** | **29** | **22** | **30** | **10** | **0.374** | **0.012** |
| **medial dorsal thalamic area** | **3** | **16** | **27** | **0** | **0.384** | **-0.016** |
| **internal capsule** | **0** | **15** | **14** | **6** | **0.385** | **0.002** |
| **dorsal medial hypothalamic area** | **27** | **22** | **0** | **0** | **0.392** | **-0.025** |
| **parafascicular thalamic area** | **0** | **10** | **0** | **0** | **0.404** | **-0.037** |
| **stria medullaris** | **0** | **0** | **0** | **0** | **0.404** | **0.005** |
| **caudate putamen** | **3** | **23** | **10** | **9** | **0.419** | **0.019** |
| **forceps minor corpus callosum** | **8** | **17** | **0** | **22** | **0.457** | **-0.002** |
| **paraventricular hypothalamic area** | **0** | **0** | **0** | **20** | **0.460** | **-0.002** |
| **lateral paragigantocellular area** | **7** | **18** | **17** | **6** | **0.463** | **-0.015** |
| **fimbria hippocampus** | **22** | **35** | **22** | **10** | **0.475** | **-0.006** |
| **medial septal area** | **0** | **23** | **0** | **0** | **0.495** | **-0.031** |
| **medial geniculate** | **0** | **7** | **15** | **2** | **0.512** | **-0.055** |
| **substantia nigra** | **2** | **6** | **27** | **10** | **0.525** | **-0.042** |
| **corpus callosum** | **11** | **10** | **13** | **16** | **0.532** | **-0.038** |
| **CA3** | **14** | **28** | **20** | **14** | **0.535** | **-0.036** |
| **locus ceruleus** | **0** | **0** | **0** | **0** | **0.538** | **0.041** |
| **lateral caudal hypothalamic area** | **3** | **9** | **17** | **0** | **0.554** | **-0.049** |
| **dorsal hippocampal commissure** | **0** | **0** | **0** | **0** | **0.632** | **-0.087** |
| **habenular area** | **5** | **27** | **43** | **0** | **0.645** | **-0.048** |
| **medial amygdaloid area** | **18** | **14** | **13** | **6** | **0.718** | **-0.069** |
| **external capsule** | **9** | **15** | **8** | **3** | **0.732** | **-0.053** |
| **retrosplenial rostral ctx** | **5** | **7** | **16** | **3** | **0.750** | **-0.071** |
| **CA1** | **14** | **24** | **18** | **10** | **0.758** | **-0.083** |
| **ventral tegmental area** | **3** | **5** | **0** | **0** | **0.768** | **-0.098** |
| **zona incerta** | **0** | **3** | **6** | **0** | **0.818** | **-0.102** |
| **lateral septal area** | **8** | **20** | **13** | **8** | **0.901** | **-0.081** |
| **parietal ctx** | **3** | **0** | **0** | **0** | **0.978** | **-0.104** |
| **reuniens thalamic area** | **6** | **0** | **0** | **0** | **0.979** | **-0.103** |
| **extended amydala** | **0** | **4** | **0** | **0** | **0.998** | **-0.150** |

**Additional file: phMRI Analysis**

The statistical significance of all changes was assessed for each voxel (~15,000 per subject, in their original reference system) with an independent Student t-tests, with a 2% threshold to account for normal fluctuations of BOLD signal in the awake rodent brain (1). As a result of the multiple t-tests performed, a false-positive detection controlling mechanism was introduced (2), to ensure that, on average, the false positive detection rate remained below 0.05. The following formula was used:

| $P_{i}\leq\frac{i}{V}\frac{q}{c\left( V \right)} ,$ | (3) |
| --- | --- |

where $P_{i}$ is the *p-*value from the t-test at the $i$-th pixel within the region of interest (ROI) containing $V$ pixels, each ranked based on its probability value. The false-positive filter value $q$ was set to 0.2 for our analysis, and the predetermined constant $c\left( V \right)$ was set to unity (3), providing conservative estimates for significance. Pixels that were statistically significant retained their relative percentage change values, while all other pixel values were set to zero. A 95% confidence level, two-tailed distributions, and heteroscedastic variance assumptions were employed for the t-tests.

Voxel-based percent changes in BOLD signal were combined across subjects within the same group to build representative functional maps. To this end, all functional images were skull stripped and co-registered (motion correction) using SPM12 (<https://www.fil.ion.ucl.ac.uk/spm/software/> spm12/) with the following parameters: Quality: 0.97, Smoothing: 0.35 mm, Separation: 0.50 mm. Gaussian smoothing was performed with a FWHM of 0.8 mm. Functional Images were first registered to a 3D Mouse Brain Atlas© with 138 segmented and annotated brain regions (Ekam Solutions; Boston, MA). The image registration involved translation, rotation, and scaling, independently and in all three dimensions. All applied spatial transformations were compiled into a matrix $\left[ T_{j} \right]$ for the $j$-th subject. Every transformed anatomical pixel location was tagged with a brain area to generate fully segmented representations of individual subjects within the atlas.

Next, composite maps of the percent changes in BOLD signal were built for each experimental group. Each composite pixel location (row, column, and slice) was mapped to a voxel of the $j$-th subject by virtue of the inverse transformation matrix ${[T_{j}]}^{-1}$. A tri-linear interpolation of subject-specific voxel values determined their contribution to the composite representation. The use of the inverse matrices ensured that the full composite volume was populated with subject inputs. The average of all contributions was assigned as the percent change in BOLD signal at each voxel within the composite representation of the brain for the respective experimental group. The number of activated voxels in each of the 138 regions was then compared between the control and CBD doses using a Kruskal-Wallis test statistic. *Post hoc* analyses were performed with a Wilcoxon rank-sum test. Brain regions were considered statistically different between experimental groups when *p* ≤ 0.05.

**References**

1. Brevard ME, Duong TQ, King JA, Ferris CF (2003): Changes in MRI signal intensity during hypercapnic challenge under conscious and anesthetized conditions. *Magn Reson Imaging*. 21:995-1001.

2. Genovese CR, Lazar NA, Nichols T (2002): Thresholding of statistical maps in functional neuroimaging using the false discovery rate. *NeuroImage*. 15:870-878.

3. Benjamini Y, Hochberg Y (1995): Controlling the false discovery rate: A practical and powerful approach to multiple testing. *Journal of the Royal Statistical Society Series B (Methodological)*. 57:289-300.

**Additional file: Resting State BOLD Functional Connectivity Analysis**

Preprocessing was accomplished by combining Analysis of Functional NeuroImages (AFNI_17.1.12, http://afni.nimh.nih.gov/afni/), FMRIB Software library (FSL, v5.0.9, http://fsl.fmrib.ox.ac.uk/fsl/), Deformable Registration via Attribute Matching and Mutual-Saliency Weighting (DRAMMS 1.4.1,https://www.cbica.upenn.edu/sbia/software/drams/index .html) and MATLAB (MathWorks, Natick, MA). Brain tissue masks for rsFC images were manually drawn using 3DSlicer (https://www.slicer.org/) and applied for skull-stripping. While FSL is routinely used in human studies to extract the brain, we find the algorithm is inadequate for rodent studies. Pre-defined cerebrospinal fluid (ventricles) and white matter regions are segmented based on the 3D MRI mouse brain atlas. After registration, the averaged time-courses were extracted and normalized. Outliers (i.e., data corrupted by extensive motion) if detected in the dataset, the corresponding time points were recorded so that they could be regressed out in a later step. Functional data were assessed for the presence of motion spikes. Any large motion spikes, if identified, were removed from the time-course signals. This motion censoring step was followed by slice timing correction from interleaved slice acquisition order. Head motion correction (six motion parameters) was carried out using the first volume as a reference image. In these studies, six of the mice (4 for vehicle and 2 for CBD) were positioned in the coil such that the field of view included the neck and apex of the lungs adding unwanted and uncorrectable motion. These mice were eliminated from the study. We did not use framewise-displacement-based scrubbing commonly used in human imaging a method that sums the absolute values of the derivatives of the six realignment parameters to identify excessive head motion. Instead, motion censoring was achieved by AFNI 3dToutcount and outliers were defined as number of MAD (median absolute deviation) that are allowed. The output contains the fraction of voxels per volume (within the automask) that exceeds the outlier limits after 3rd degree legendre polynomial detrending. These time points are fed into AFNI 3dDeconvolve for nuisance regression.

Normalization was completed by registering functional data to the 3D MRI rat brain atlas using affine registration through DRAMMS (1-3). The same atlas was used for segmentation into 138 brain areas. Nonlinear registration was performed to register individual images to standard rat brain template. Then, regressors comprised of demeaned motion parameters, white matter, and cerebrospinal fluid time series were fed into general linear models for nuisance regression to remove unwanted effects. Band-pass filtering (0.01Hz ~ 0.1Hz) was performed to reduce low-frequency drift effects and high-frequency physiological noise for each subject. The resulting images were further spatially smoothed with full width at half maximum set at 0.8 mm. The region-to-region functional connectivity method was performed in this study to measure the correlations in spontaneous BOLD fluctuations. A network is comprised of nodes and edges; nodes being the brain region of interest (ROI) and edges being the connections between regions. Voxel time series data were averaged in each node based on the residual images using the nuisance regression procedure with motion parameters and mean time courses of white matter and ventricles. To acquire regional temporal correlations, Pearson’s correlation coefficients were measured among (N * (N-1))/2 region pairs. For statistical inference on significant correlation, FDR (q=0.1) was applied for multiple correction. Pearson’s correlation coefficients across all pairs of nodes (9,453 pairs) were computed for each subject among all three groups to assess the interregional temporal correlations. The r-values (ranging from -1 to 1) were z-transformed using the Fisher’s Z transform to improve normality. 138 x 138 symmetric connectivity matrices were constructed with each entry representing the strength of edge. Group-level analysis was performed to look at the functional connectivity in all experimental groups. The resulting Z-score matrices from one-group t-tests (against 0) were clustered using the K-nearest neighbors (KNN) method to identify clusters of nodes that form resting state networks reordering the brain areas to give symmetrical correlation matrices. The KNN algorithm generates an automatic clustering solution which reorders the nodes based on the distance between edge information so that smaller clusters are formed along the diagonal line of the matrix. A Z-score threshold of |Z|=2.3 was applied to remove spurious or weak node connections for visualization purposes. |Z|<2.3 equates to p<0.02145 for two-tailed hypothesis (connectivity significantly greater or less than 0). With an uncorrected p value = 0.001, an accepted threshold voxel-wise analysis, the corresponding Z is 3.09.

1. Honeycutt JA, Demaestri C, Peterzell S, Silveri MM, Cai X, Kulkarni P, et al. (2020): Altered corticolimbic connectivity reveals sex-specific adolescent outcomes in a rat model of early life adversity. *Elife*. 910.7554/eLife.52651.PMC7010412.

2. Kulkarni P, Morrison TR, Cai X, Iriah S, Simon N, Sabrick J, et al. (2019): Neuroradiological Changes Following Single or Repetitive Mild TBI. *Frontiers in systems neuroscience*. 13:34 10.3389/fnsys.2019.00034.PMC6688741.

3. Ferris CF, Cai X, Qiao J, Switzer B, Baun J, Morrison T, et al. (2019): Life without a brain: Neuroradiological and behavioral evidence of neuroplasticity necessary to sustain brain function in the face of severe hydrocephalus. *Scientific reports*. 9:16479 10.1038/s41598-019-53042-3.PMC6848215.

**Additional file: Graph Theory Analysis**

*Degree centrality*

Degree centrality analysis quantifies the number of connections a specific node has to the overall network. Degree centrality is defined as:

$$C_{D}(j)=\sum_{j=1}^{n} A_{\mathrm{ij}}$$

where n is the number of rows in the matrix in the adjacency matrix **A** and the elements of the matrix are given by A_ij_, the number of edges between nodes i and j.

*Statistics*

All statistical analysis for the graph theory analysis was performed using GraphPad Prism version 9.0.0 (86) for macOS, GraphPad Software, San Diego, California USA, www.graphpad.com. Normality tests between group subregions performed to determine if parametric or non-parametric assumptions were needed. Shapiro-Wilk’s tests were performed to analyze normality assumption. Subregion degree centrality p-values that were greater than 0.05 were assumed to be normal. After assumptions of normality were validated, paired t-tests were used to compare degree centrality of the CBD and vehicle groups in various subregions. When necessary, a nonparametric Wilcoxan signed rank (WSR) test is performed if there is evidence against the normality assumption.

*Visualization of the ARAS connections to the olfactory system following CBD treatment*

Differences in degree between CBD and control groups were visualized using Gephi software. Nodes that had a higher degree centrality in CBD than treatment were colored red and nodes that had a higher degree centrality in control were colored blue. The size of the node is scaled to reflect the relative difference in degree centrality between groups. The ARAS was designated as the following: dorsal raphe, gigantocelllaris reticular area, lateral paragigantocellular area, locus ceruleus, mesencephalic reticular formation, parabrachial area, parvicellular reticular area, pedunculopontine tegmental area, pontine reticular nucleus (caudal), and the pontine reticular nucleus (oral). The neighboring nodes of the ARAS of the CBD treated group were highlighted prior to merging into a single node. For visualization purposes, the weight of the edges was fixed and the network model was organized in a dual circle layout, displaying the olfactory system’s neighboring nodes (outside circle) with the ARAS (inside, colored grey) of the CBD treated group, with self-loops filtered.
